# Mesoporous Silicon Microspheres Produced from In Situ Magnesiothermic Reduction of Silicon Oxide for High-Performance Anode Material in Sodium-Ion Batteries

**DOI:** 10.1186/s11671-018-2699-7

**Published:** 2018-09-10

**Authors:** Dan-Feng Qiu, Xiao Ma, Jing-Dong Zhang, Zi-Xia Lin, Bin Zhao

**Affiliations:** 1 Key Laboratory of Radar Imaging and Microwave Photonics (Nanjing Univ. Aeronaut. Astronaut.), Ministry of Education, College of Electronic and Information Engineering, Nanjing University of Aeronautics and Astronautics, No. 29 Yu Dao Street, Nanjing, 210016 China; 2 National Laboratory of Microstructures and School of Electronic Science and Engineering, Nanjing University, No. 22 Hankou Road, Nanjing, 210093 China

**Keywords:** Sodium-ion battery, Mesoporous silicon, Magnesium thermal reduction, Silicon-based anode materials

## Abstract

Sodium-ion batteries have been widely used in energy storage owing to its high sodium content and low cost. This study proves that mesoporous silicon microspheres (MSMs) with the homogeneously distributed mesopores ranging from 1 to 10 nm can be used as anodes of NIBs. In situ magnesiothermic reduction of silicon oxide was carried out to synthesize the MSM samples. An anode in NIBs was tested, and it was observed that the MSMs sample which was calcined at 650 °C had a good rate performance of 160 mAh g^−1^ at 1000 mAg^−1^ and a high reversible capacity of 390 mAh g^−1^ at 100 mAg^−1^ after 100 cycles. Moreover, its long-term cycling performance was 0.08 mAh g^−1^ decay per cycle for 100 cycles, which was quite excellent. MSMs have high reversibility, good cycling performance, and excellent rate capability, which are related to its ultrafine particle size and mesoporous morphology.

## Background

Lithium-ion battery is the first choice for portable electronic equipment and electric vehicle to store energy owing to its high energy density. However, the high cost, limited resources, and uneven ground distribution of lithium are the major problems encountered in the development of grid-scale power storage systems. Because of the low cost and high abundance of Na, room-temperature sodium-ion batteries with sodium ions as the carrier of energy is one of the most promising substitutes for lithium-ion batteries (LIBS) [1–5]. However, a new design concept of electrode materials should be developed because Li^+^ (0.69 Å) and Na^+^ (0.98 Å) are different in ionic radius [6, 7]. For instance, due to the large ionic radius of Na, it is impossible to uptake a large amount of Na in the interlayer space by electrode material of commercial graphite for anodes in LIBs with the theoretical capacity of 372 mAh g^−1^. The Na–Si phase diagram [8, 9] and the prediction by Ceder and Chevrier [10] and Chou et al. [11] point out that the most Na-rich phase for Na–Si binary compounds is NaSi when Si is used as an anode in sodium-ion batteries (NIBs), so that the theoretical capacity is 954 mAh g^−1^, and Si can be a promising material for Na-ion battery anodes. The experiments also studied electrochemical sodiation [12–16] of micrometer-sized Si [17] and nanosized Si (100 nm) [18]. Mulder use Si nanoparticles as an anode in NIBs, specific capacity is about 300 mAh g ^− 1^ after 100 circles [9]. And Mukhopadhyay studied the Crystalline core/amorphous shell-structured silicon nanowires specific capacity as high as 390 mAh g^−1^ after 200 circles [19]. Since amorphous Si is conductive to the insertion of Na and nanoscale is favorable for the insertion and extraction kinetics of ions, Si particles with smaller size and large fraction of amorphous Si obtained by expanding silane was thoroughly explored [20, 21].

However, the high cost and complicated synthesis of the compounding methods may make it difficult to realize large-scale production. Therefore, it is quite urgent to develop an efficient and simple method to synthesize Si anode material with good performance [22–24]. By using in situ magnesiothermic reduction of silicon oxide, mesoporous silicon microspheres (MSMs) with diameters ranging from 1 to 10 nm homogeneously distributed in the silicon microspheres was studied. The experimental results show that reversible electrochemical Na-ion absorption can be achieved in Si, and remarkable capacity is obtained. Transmission electron microscopy (TEM), scanning electron microscopy (SEM), and X-ray diffraction (XRD) were used to characterize the final product, which was further evaluated through cycling test. When the current density was increased to 1000 mAg^−1^, more than 40% of the capacity can be retained through NIBs; thus, the microspheres are used as anode materials.

## Methods/Experimental

The modified Stöber process was used to synthesize SiO_2_ microspheres. Twenty milliliters of tetraethyl orthosilicate was added to 100 mL deionized H_2_O. Twenty milliliters of NH_3_·H_2_O and 80 mL 2-propanol were added to the mixture and magnetically stirred at the room temperature. After the reaction lasted for 2 h, the colloidal SiO_2_ spheres were collected through centrifugation, washed through deionized water and ethanol, and dried at 100 °C. Five hundred sixty milligrams of as-prepared SiO_2_ microspheres, and 600 mg magnesium powder were put in two stainless steel containers separately. Afterwards, the containers were put in a sealed stainless steel oven and heated at 650 °C for 2 h under Ar protection. The reaction mechanism is as below:

1 $$ 2\mathrm{Mg}+{\mathrm{SiO}}_2\to \mathrm{Si}+2\mathrm{Mg}\mathrm{O} $$

The magnesium compounds and the remaining magnesium were dissolved by storing the brown-yellow powder in 1 M hydrochloric acid (HCl) solution (200 ml, 1 M) for 12 h. The mixture was filtered through distilled water, and the powder was dried in under vacuum for 12 h at 80 °C. Si microsphere powder was purchased from Sigma-Aldrich Co. LLC for further comparison. 2032-type coin cells were used to carry out electrochemical measurements. A slurry was formed by adding the polyvinyldifluoride (10 wt.%), acetylene black (20 wt.%), and active material (70 wt.%) to *N*-methylpyrrolidone. Doctor blading method was adopted to paste the slurry on a copper foil current collector, which was dried under vacuum to the final weight of 2 mg/cm^2^. We assembled the half-cell Na-ion batteries in an Ar-filled glovebox with Celgard2250 as separator, 1 M NaClO_4_ dissolved in an ethylene carbonate, and diethyl carbonate mixture (1:1 by volume) as electrolyte, Na foil as counter electrode, and MSMs as working electrode. Galvanostatic charge and discharge experiments of the cells were carried out on a battery test system (LAND, Wuhan Jinnuo Electronics Ltd.) at different current densities from 0.01–2.5 V.

## Results and Discussion

**Fig. 1:**
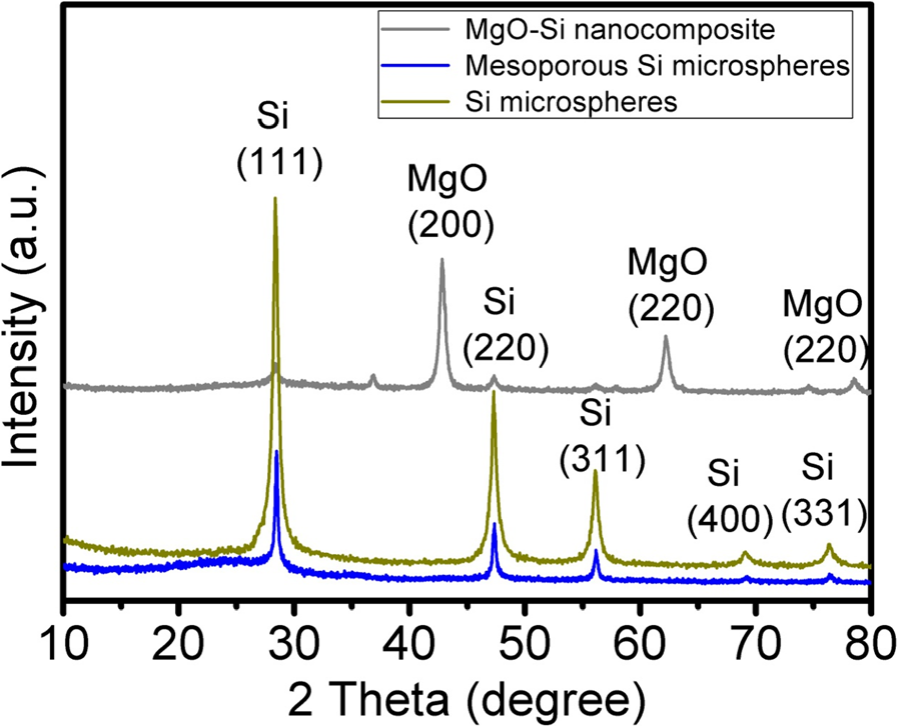
XRD patterns of the samples

The XRD patterns of the as-formed MgO–Si nanocomposite, MSMs, and the Si microspheres are shown in Fig. 1. The main diffraction peaks at 2*θ* = 28.4°, 47.4°, 56.2°, 69.2°, and 76.4° presented by MSMs can be indexes as (1 1 1), (2 2 0), (3 1 1), (4 0 0), and (3 3 1) planes of Si crystallites (JCPDS 772107). There was no extra peak relevant with the impurity in the XRD patterns. HCl solution could completely wash MgO in the MgO–Si nanocomposite.

**Fig. 2:**
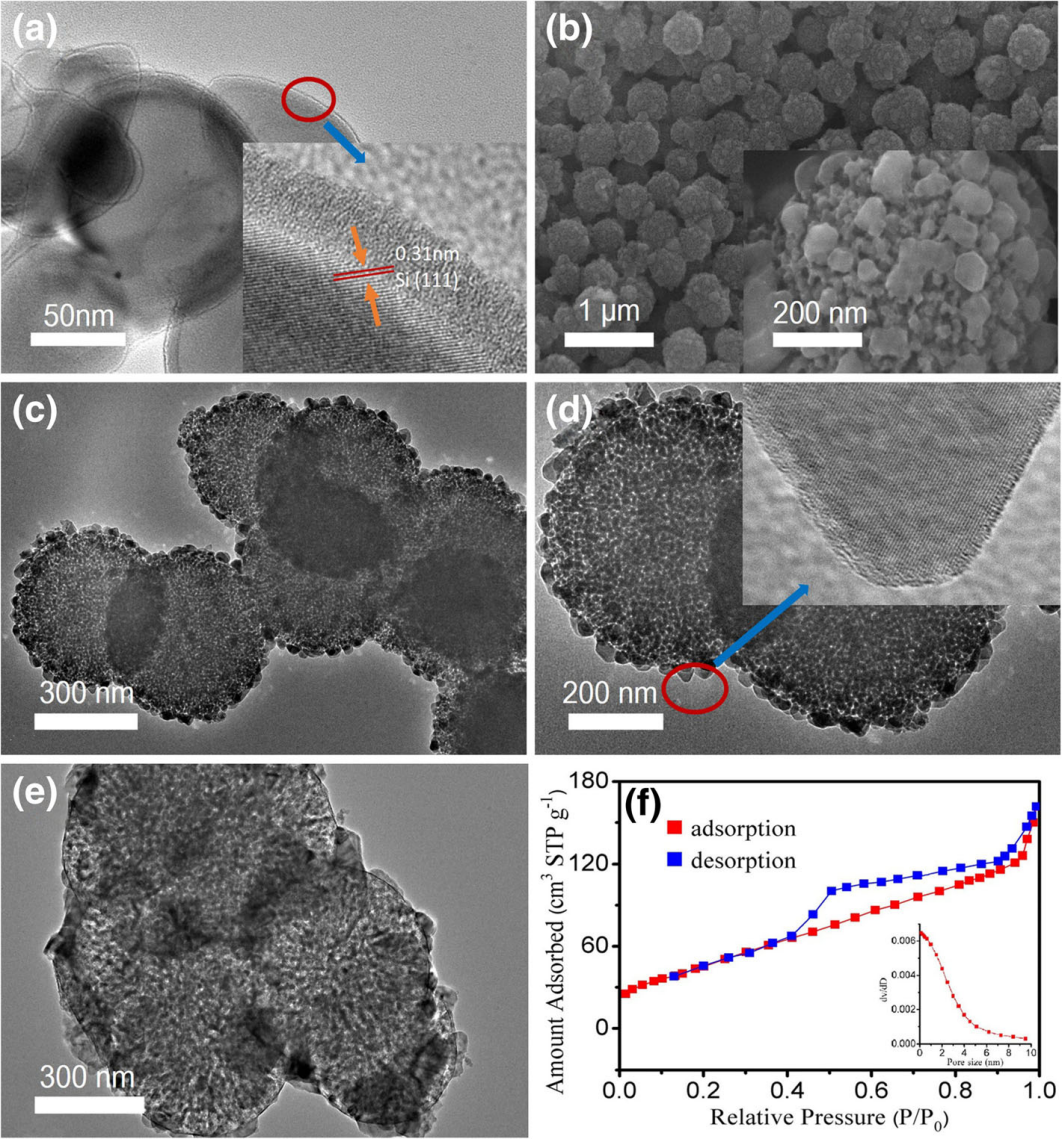
SEM (**a**) and inset TEM (**a**) images of silicon microspheres. SEM (**b**) and TEM (**c** and **d**) images of MSMs. TEM (**e**) 100 after circles in 100 mAg^− 1^. The adsorption–desorption curve (**f**) of MSMs, inset: particle size distribution of MSMs

SEM and TEM were adopted to examine the morphologies of mesoporous Si microspheres and Si microspheres. The typical TEM and SEM images of the Si sphere are shown in Fig. 2a. The SEM images of mesoporous Si microspheres at various magnifications are shown in Fig. 2b. There are abundant mesopores in Si microspheres. The TEM images of MSMs are shown in Fig. 2c, d. The diameters of the mesoporous structure of Si microspheres are from 1 to 10 nm. Figure 2e is the TEM image of the MSMs circles at the density of 100 mAg^−1^. A typical type-IV isotherm with a type H3 hysteresis loop can be observed in the adsorption-desorption curve (Fig. 2f), which indicates the disordered mesopores in MSMs. According to the Barrett–Joyner–Halenda (BJH) pore size distribution curve from the adsorption branch, the pore distribution is below 6 nm, which is accordance with the TEM result. The pore volume and BET surface area were 0.25 cm^3^ g^−1^ and 200 m^2^ g^−1^. Since the mesopores serve as buffer zone, the volume variations of silicon are accommodated effectively by MSMs which can maintain the structure in charging and discharging process. Good electronic conductivities can be maintained by adding conductive carbon, which is conductive to the electrode materials in NIBs.

**Fig. 3:**
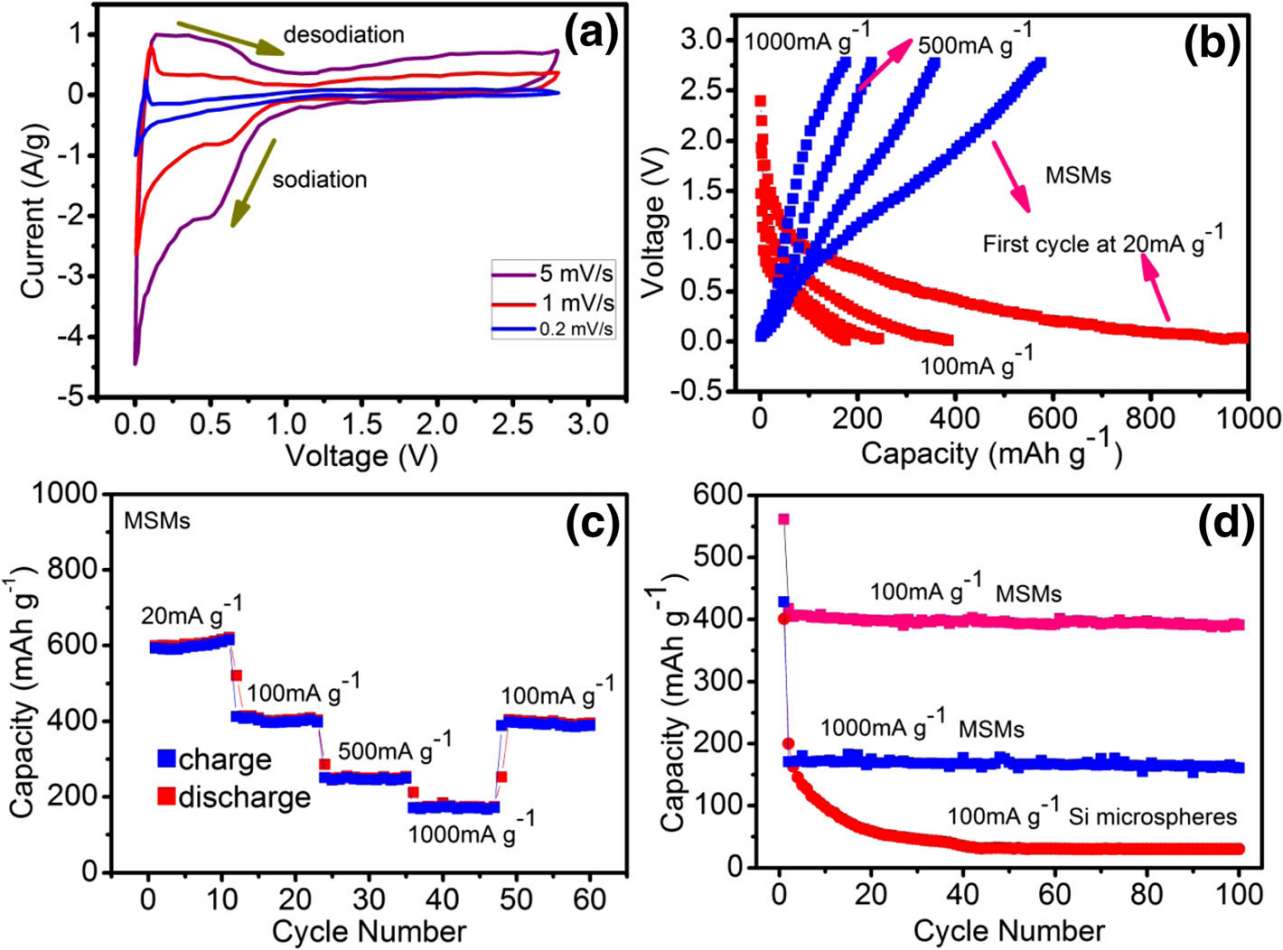
**a** Cyclic voltammetry measurements of MSMs at different current density, **b** representative charge–discharge curves of MSMs at various current densities, **c** capacity retention of MSMs at various current densities, and (**d**) discharge capacity retention of MSMs and silicon nanospheres at a current density of 1000 mAg^−1^ and 100 mAg^−1^

We carried out cyclic voltammetry (CV) measurements from 0.01 to 2.5 V at various scan rates. As shown in Fig. 3a, when the scan rate is 0.2 mV s^−1^, there is an obvious cathodic peak at 0.04, which can be attributed to the insertion of Na ion to crystalline Si. The crystalline Si is extracted at 0.08 V through the anodic scan. Na absorption in amorphous Si occurs in a wider and higher voltage range (< 0.8 V) [9]. With the increase of scanning rate, the potential peak gradually shifts to the lower alkali voltage and higher decarbonization potential, which is caused by the increasingly significant overpotential. Figure 3b shows the typical charge–discharge curves of mesoporous Si microspheres at the current densities from 0.01 V and 2.5 V versus Na^+^/Na. The formation of NaSi causes the plateau at 0.6 V in the first discharge curve. The increase of current density leads to the decrease of the discharge potential and the increase of the charge potential of MSMs. As a result, high overpotentials occur. The cell was cycled for 10 cycles at the low current density of 100 mAg^−1^, and the stable specific capacity was about 400 mAh g^−1^. The proportion of the retained capacity is greater than 40% at 1000 mAg^−1^, which indicates the excellent rate capability of MSMs. After 60 charge–discharge cycles, the capacity of approximately 390 mAh g^−1^ was retained at different current densities (Fig. 3c). Therefore, the cycling stability is good. The charge/discharge capacity curves of the electrodes which are made of MSMs versus the cycle number at the charge–discharge current density of 100 mAg^−1^ at 25 °C are shown in Fig. 3d. The capacity of silicon for the first charge and discharge of sodium-ion batteries is larger than that for the second charge and discharge, which is mainly due to the irreversible sodium-ion intercalation and SEI film formation during the first charge and discharge. After 100 cycles, the capacity is about 390 mAh g^−1^, and the MSMs electrode has an excellent long-term cycling performance of 0.08 mAh g^−1^ decay per cycle, which indicates the good cyclic stability of the electrode. As for pure Si microspheres, the electrode only retained 30 mAh g^−1^ after 100 cycles under the charge–discharge current density of 100 mAg^− 1^. The cycling stability of MSMs was enhanced.

**Fig. 4:**
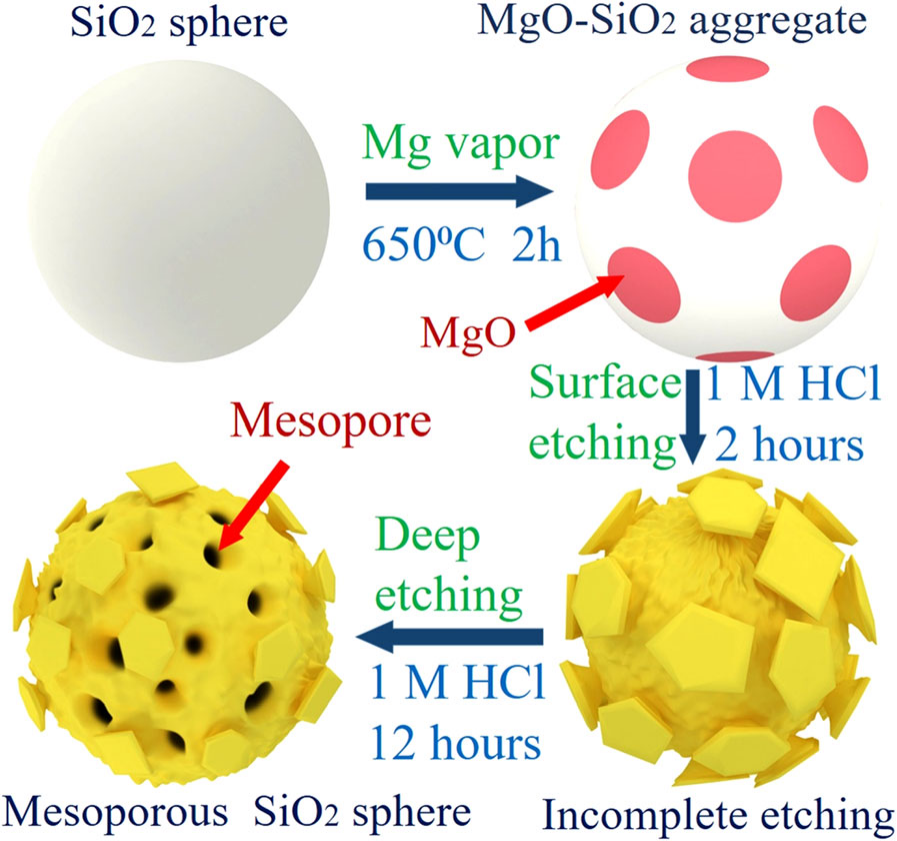
Schematic illustration of the MSMs

Figure 4 shows the typical synthesis process of MSMs. Silica microspheres have large specific surface area and can be regarded as a suitable silicon source. Therefore, MSMs were synthesized by using Si microspheres as silicon source in the magnesiothermic reduction process. The molten magnesium vapor reacts in silica microspheres and forms MgO–Si nanocomposite at 650 °C. MgO is further removed by treating nanocomposite through HCl solution in the etching process. 3D MSMs are formed by the residual silicon nanocrystals, and the volume variations of silicon during the repeated alloying and de-alloying cycles are accommodated by taking the well-dispersed mesopores as a buffer zone. The exfoliation and aggregation of Si particles are suppressed. Both crystalline and amorphous Si play an active role in electrochemical alkalization. NaSi and Si may coexist when Na is inserted to amorphous Si and Si crystallites. When Na is extracted, the solid solution deoxidation reaction is confirmed. Mesoporous also provides a useful electrolyte channel for sodium-ion transfer, which explains the improvement of electrochemical performance of MSMs.

## Conclusions

The magnesiothermic reduction method was used to prepare a 3D mesoporous silicon material. The research results demonstrate that reversible electrochemical Na-ion absorption can be realized at room temperature. This improvement can be attributed to optimized nanostructures relevant with the uniformly distributed mesoporous structures.

## Abbreviations

3D: Three dimensions
BJH: Barrett–Joyner–Halenda
CV: Cyclic voltammetry
HCl: Hydrochloric acid
LIBs: Lithium-ion batteries
MgO: Magnesium oxide
MSMs: Mesoporous silicon microspheres
NaSi: Sodium silicide
NIBs: Sodium-ion batteries
SEM: Scanning electron microscopy
Si: Silicon
TEM: Transmission electron microscopy
XRD: X-ray diffraction
